# Arc/Arg3.1 protein expression in dorsal hippocampal CA1, a candidate event as a biomarker for the effects of exercise on chronic stress-evoked behavioral abnormalities

**DOI:** 10.20463/jenb.2017.0033

**Published:** 2017-12-31

**Authors:** Yea-Hyun Leem, Hyukki Chang

**Affiliations:** 1. Exercise Physiology Laboratory, Department of Human Movement Science, Seoul Women's University, Seoul, Republic of Korea.

**Keywords:** Arc, chronic stress, memory consolidation, depression, regular exercise, AMPA receptor

## Abstract

**[Purpose]:**

Chronic stress is a risk factor for behavioral deficits, including impaired memory processing and depression. Exercise is well known to have beneficial impacts on brain health.

**[Methods]:**

Mice were forced to treadmill running (4-week) during chronic restraint stress (6h/21d), and then behavioral tests were conducted by Novel object recognition, forced swimming test: FST, sociality test: SI. Dissected brain was stained with anti-calbindin-d28k and anti-Arc antibodies. Also, mice were treated with CX546 intraperitoneally during chronic restraint stress, and behavioral tests were assessed using Morris water maze, FST, and SI. Dissected brain was stained with anti-Arc antibody.

**[Results]:**

The current study demonstrated that chronic stress-induced impairment of memory consolidation and depression-like behaviors, along with the changes in calbindin-d28k and Arc protein levels in the hippocampal CA1 area, were attenuated by regular treadmill running. Further, prolonged ampakine treatment prevented chronic stress-evoked behavioral abnormalities and nuclear Arc levels in hippocampal CA1 neurons. Nuclear localization of Arc protein in hippocampal CA1 neurons, but not total levels, was correlated with behavioral outcome in chronically stressed mice in response to a regular exercise regimen.

**[Conclusion]:**

These results suggest that nuclear levels of Arc are strongly associated with behavioral changes, and highlight the role of exercise acting through an α-amino-3-hydroxy-5-methyl-4-isoxazolepropionic acid (AMPA) receptor (AMPAR)-mediated mechanisms in a chronic stress-induced maladaptive condition.

## INTRODUCTION

Chronic stress is a potent predictor for the development of memory processing impairments and psychiatric defects, including longterm memory impairments and depression. In particular, chronic stress-evoked abnormalities in activity-dependent synaptic plasticity are strongly associated with these neuropathophysiological conditions ^1^. 

The activity-regulated cytoskeletal gene Arc, also known as Arg3.1, plays a critical role in synaptic strength and plasticity during memory consolidation. Its transcription, translation, localization, and stability are tightly controlled by neuronal activity ^2^, suggesting various functions depending on its particular characteristics. In fact, Arc plays a pivotal role in long-term potentiation (LTP) by regulating cytoskeletal dynamics and spine morphology, as well as in long-term depression (LTD) by mediating metabotropic glutamate receptor (mGluR)-induced LTD via α-amino-3-hydroxy-5-methyl-4-isoxazolepropionic acid (AMPA) receptor (AMPAR) endocytosis at synapses ^3^^-^^4^. Several studies have demonstrated that Arc expression mostly increases in response to acute stress in the prefrontal cortex, with an AMPAR and N-methyl-D-aspartate (NMDA) receptor (NMDAR)-dependent increase in glutamatergic neurotransmission ^5^^-^^6^. In particular, cortical AMPAR expression and AMPAR-mediated excitatory postsynaptic currents (EPSCs) are induced by various acute stressors ^6^^-^^7^. Specific chronic stress-induced changes of hippocampal Arc expression remain unclear; conflicting studies have shown that hippocampal Arc expression is either increased or decreased by chronic stress ^8^^-^^9^. Arc protein localizes to active synapses and nuclei, though its function has been extensively explored at the synapse. Translated Arc protein translocates into the nucleus, and contributes to GluR1 expression and synaptic plasticity in stimulated neurons, in in vivo and in vitro experiments ^10^^-^^11^. 

Various forms of exercise have the ability to prevent, restore, or ameliorate chronic stress-induced brain disorders, including cognitive impairment and depression ^12^^-^^13^. For example, chronic stress-induced impairments in learning and memory are ameliorated by regular exercise via cAMP-activated protein kinase (AMPK)-dependent BDNF activity in chronically stressed mice ^12^. Chronic stress-induced abnormalities in glutamate transmission and synaptic plasticity are associated with AMPAR function, as evidenced by a decrease in excitation of temporoammonic (TA)-CA1 path synapses, and a decrease in AMPAR expression in the hippocampal CA1 region ^13^^-^^14^. On the contrary, 4 weeks of voluntary wheel running, (but not acute exercise) enhances GluR1 and pGluR1 (Ser845) levels in the hippocampus ^15^. Repeated exercise alters AMPAR subunit distribution in diverse brain regions, evidenced by changes in AMPAR subunits in the sensory-motor cortex, cerebellum, and striatum ^16^. 

However, the nuclear localization of Arc protein in hippocampal CA1 neurons, the behavioral outcomes of chronic stress, and the effects of regular exercise on such factors are under-explored. The present study demonstrates a correlation between chronic stress-induced alterations in nuclear Arc levels in hippocampal CA1 neurons and AMPAR activity, and explores the potential role of exercise. 

## METHODS

### Experimental mice

Male 7-week-old C57BL/6 mice were obtained from Dae Han Biolink Co., Ltd. (Eumsung, Chungbuk, Korea), and all experimental procedures involving animals were approved by the Animal Care and Use Committee at Seoul Women’s University. 

### Behavioral tests

Depressive-like behavior (N=7-8/group) was assessed using two methods: the forced swimming test (FST) and the sociality test ^17^. For the FST, all mice were exposed to a 15-min pre-test on day 1. The test was then conducted 24 h later and recorded using a video camera. Mice were forced to swim for 6 min, and the time spent immobile was measured after the first min. Briefly, for the sociality test, an apparatus was partitioned into three equal chambers with clear Plexiglas dividing walls that could be removed to allow free access to each chamber. Before testing, each mouse was first acclimatized by being placed into the closed-off center compartment for 5 min. An unfamiliar conspecific male mouse that had no prior contact with the test mouse was enclosed in a wire cup in either the third spot from the left or the right chamber for 1 min, following which the test mouse was allowed to explore for 5 min by removing both dividing walls. Sociality was quantified as the time spent in the interaction zone (IZ) with the cup containing the novel male, or the non-interaction zone (Non-IZ) containing an empty cup. Social interaction (SI) index was calculated using the following equation: SI (%) = IZ/(non-IZ + IZ) x 100. 

Memory consolidation was evaluated using two methods: the Morris water maze (MWM) ^18^ and the novel object recognition test ^19^. For the MWM, mice were randomly assigned a quadrant (NW, NE, SE, or SW) located in a 1.5-m-diameter black circular pool containing 22°C water that contained a stationary escape platform. Probe tests were conducted 24 h after the last 10-trial training block as well as 28 days later, wherein the platform was removed and mice swam from a random start location for 60 s. The time spent in the target quadrant (that used to contain the platform) and the latency to the target were determined using SMART 3.0 software (Panlab, S.L.U., Barcelona, Spain) on a computer connected to a ceiling-mounted camera directly above the pool. For the novel object recognition test, mice were handled and habituated to an empty open field arena for 2 days. An acquisition session was performed, wherein mice were placed into the arena with two identical objects (Falcon tissue culture flasks filled with sand) that were positioned in two corners of the apparatus, and allowed to explore the objects for 10 mins. Following a 3 day delay, one of the familiar objects was replaced with a novel object (interlocking tower of Lego pieces with different shapes and colors), and mice were allowed to explore for 5 mins. The time spent exploring each object was recorded using a video-tracking system during the acquisition and retention phases. The recognition ratio (%) was expressed as the percentage of time spent with each object compared with the total exploration time. 

To facilitate sustained AMPAR activation, CX546 [0-20 mg/kg CX546 dissolved in 16.5% 2-hydroxypropyl-b-cyclododextrin (CDX) in 0.9% saline, Abcam, Cambridge, UK], a potent ampakine, was injected intraperitoneally twice daily every 2 days during the period of stress exposure, a dose regimen chosen based on preliminary work wherein this paradigm increased BDNF protein levels without altering exploratory behavior ^20^. 

### Immunohistochemical analyses

Anesthetized mice were perfused with 100 mM PBS (pH 7.4), followed by cold 4% paraformaldehyde in PBS. Every fourth section (40 μm-thickness) was taken from the region between bregma -1.82 mm and -2.18 mm. Free-floating staining was conducted using traditional methods. Sections were incubated overnight at 4°C with anti-calbindin-D-28k (CalB) and anti-Arc/Arg3.1 antibody (Abcam, Cambridge, rabbit polyclonal, 1: 2,000) then with biotinylated secondary antibodies (Vector Laboratories; Burlingame, CA, USA 1: 200, respectively), followed by visualization using the ABC and DAB methods (ABC Elite kit, Vector Laboratories; Burlingame, CA, USA). Sections were slide mounted and digitally imaged (captured at 100× magnification), then analyzed using Image J software (NIH Image Engineering, Bethesda, MD). The number of positive cells per mm2 was counted within a defined circle (each group N=4-5). 

### Statistical analyses

Significant differences between groups were determined using paired t-tests and one-way analyses of variance (ANOVA; SPSS for Windows, version 18.0, Chicago, IL, USA). Post-hoc comparisons were performed using Student-Newman-Keuls tests. All values are reported as mean ± standard deviation (SD). Values of p < 0.05 were considered statistically significant. 

## RESULTS

Regular exercise prevented chronic stress-induced failure of memory consolidation and behavioral depression, with a simultaneous change in calbindin levels as well as total and nuclear Arc protein levels in hippocampal CA1 neurons. 

**Figure 1.**
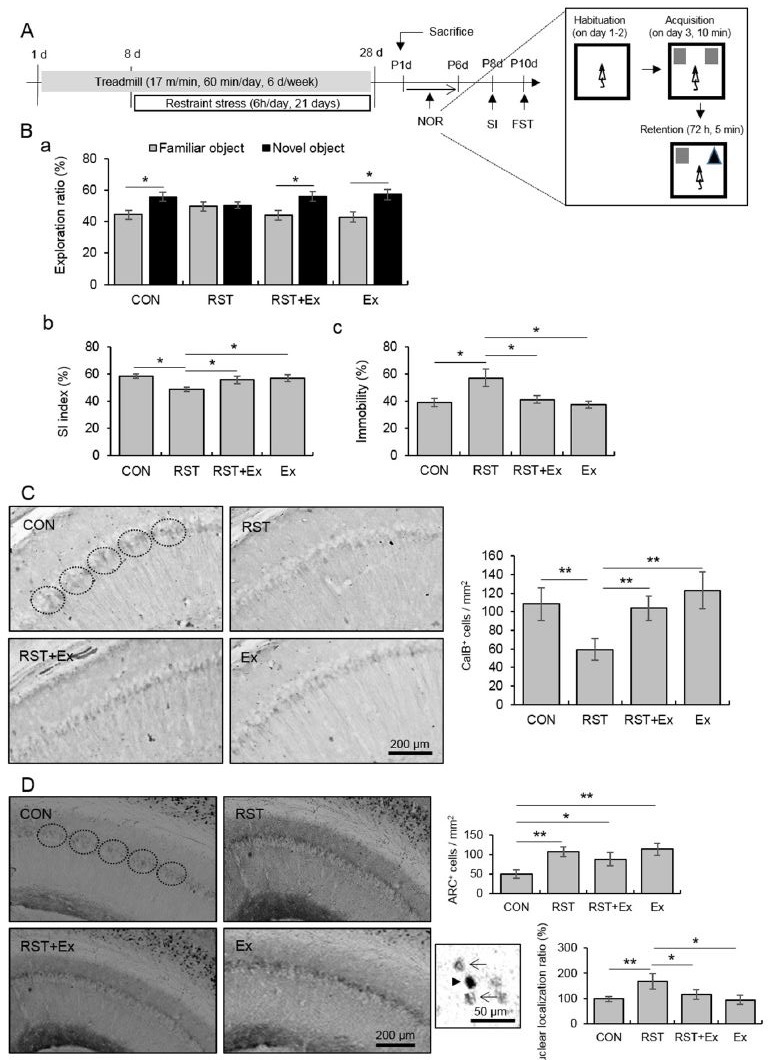
Regular exercise protected against chronic stress-induced impairments in memory consolidation and the development of behavioral depression, along with changes in CalB and Arc protein levels in hippocampal CA1 cells. **A. ** Experimental procedures (CON: control; RST: restraint stress; RST+Ex: restraint stress+treadmill running, Ex: treadmill running). **B. ** Quantitative analysis of memory consolidation, measured by the novel object recognition test (a), the social interaction index measured by the sociality test (b), and immobility measured by the forced swimming test (c). **C. ** Quantitative analysis of CalB+ cells. Photomicrograph showing immunohistochemistry (right panel) and quantification (left panel). **D. ** Quantitative analysis of Arc protein levels. Photomicrograph showing immunohistochemistry (upper panel), total Arc+ cells (left upper pan el), and nuclear ratio (right bottom panel). Arrow head denotes nuclear localization and arrow denotes cytoplasmic localization. Data are presented as means ± SD (n = 10-12 animals). * and ** denote significant differences at *p* < 0.05 and *p* < 0.01, respectively.

We found that chronic stress (6h/21d) induced a decrease in the exploration ratio of the novel object, a decrease in SI index, and an increase in immobility during the FST, which was reversed by regular exercise (Fig. 1AB; for the exploration ratio: CON t12 = -2.56, p <0.05; RST t14 = -0.02, p > 0.05; RST+Ex t14 = -2.67, p < 0.05; Ex t12 = -2.79, p < 0.05; for SI: F3, 26 = 3.03, p < 0.05; for immobility: F3, 26 = 4.13). 

There were fewer CalB+ hippocampal CA1 cells as a result of chronic stress, and this decrease was restored to control levels by exercise (Fig. 1C; F3, 12 = 12.12, p < 0.01). There was a profound enhancement of Arc+ hippocampal CA1 cells following chronic stress, regardless of treadmill running or exercise alone regimens (Fig. 1D; F3, 12 = 11.99, p < 0.01). Chronic stress-induced enhancement of the ratio of nuclear to cytoplasmic levels of Arc was restored to basal levels by exercise, and the nuclear ratio of Arc in exercise alone mice was comparable to that of controls (Fig. 1D right panel; F3, 12 = 8.54, p < 0.01). 

Prolonged ampakine treatment prevented chronic stress-induced failure of memory consolidation and behavioral depression, with a simultaneous change in total and nuclear Arc protein levels in hippocampal CA1 neurons. 

To facilitate sustained AMPAR activation, CX546 [0-20 mg/kg CX546 dissolved in 16.5% 2-hydroxypropyl-b-cyclododextrin (CDX) in 0.9% saline], a potent ampakine, was injected intraperitoneally twice daily every 2 days during the period of stress exposure. 

**Figure 2.**
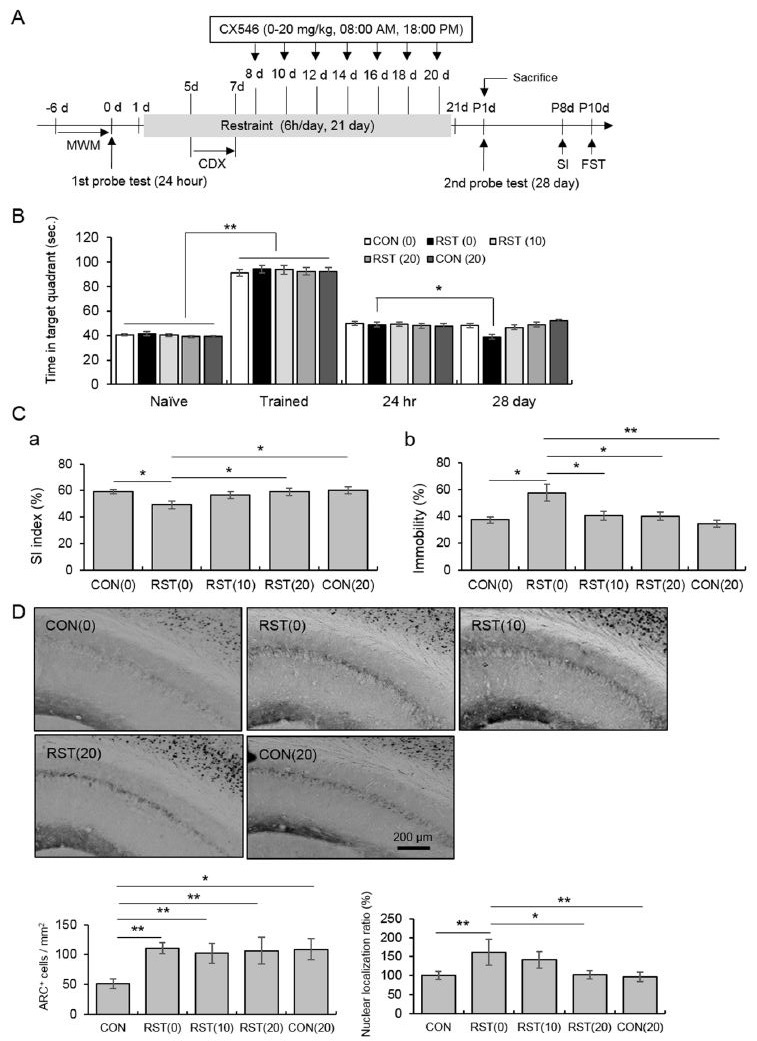
CX546 protected against chronic stress-induced failure of memory consolidation and development of behavioral depression, along with changes of Arc protein levels on hippocampal CA1 area. **A. ** Experimental procedures. **B. ** Quantitative analysis of long-term memory measured by the novel object recognition test. **C. ** Quantitative analysis of the social interaction index measured by the sociality test (a), and immobility measured by the forced swimming test (b). **D. ** Quantitative analysis of Arc protein levels. Photomicrograph showing immunohistochemistry (upper panel), total Arc+ cells (left upper panel), and nuclear ratio (right bottom panel). Arrow head denotes nuclear localization and arrow cytoplasmic localization. Data are presented as means ± SD (n = 10-12 animals). * and ** denote differences at *p* < 0.05 and *p* < 0.01, respectively.

A 10-block training paradigm was used in the MWM, which resulted in the successful consolidation of longterm memory (Fig. 2A-B; CON: t12 = -19.36, p < 0.01; RST(0): t12 = -17.42, p <0.01; RST(10): t12 = -17.01, p < 0.01; RST(20): t12 = -19.13, p < 0.01; CON(20): t12 = -16.30, p < 0.01). Time spent in the target quadrant was significantly reduced by chronic stress, and was returned to basal levels following treatment with CX546, 28 days after the last exposure of restraint (Fig. 2A-B; CON: t12 = 1.32, p > 0.05; RST(0): t12 = -2.38, p <0.05; RST(10): t12 = 1.76, p > 0.05; RST(20): t12 = 0.42, p > 0.05; CON(20): t12 = -5.80, p > 0.01). In the sociality test, chronic stress reduced the SI index, and this decrease was attenuated by CX546 administration (20 mg/kg; Fig. 2Ca; F4, 30 = 2.63, p < 0.05). Immobility in the FST was enhanced by chronic stress, and this increase was reversed by CX546 (10-20 mg/kg; Fig. 2Cb; F4, 30 = 4.98, p < 0.01). The immunoreactivity of Arc in hippocampal CA1 cells was profoundly enhanced by chronic stress, regardless of CX546 treatment (Fig. 1D; F4, 20 = 10.31, p < 0.01). Chronic stress-induced enhancement of the nuclear localization ratio of Arc (i.e., the ratio of nuclear to cytoplasmic levels) returned to basal level following CX546 treatment (20 mg/kg; Fig. 1D right panel; F4, 20 = 8.25, p < 0.01). 

## DISCUSSION

The current study demonstrated that regular exercise exerted protective effects against chronic stress-induced behavioral deficits, including the impairment of longterm memory formation and the development of depressive symptoms, likely by reducing AMPAR-mediated excitatory responsiveness in hippocampal CA1 cells. 

The 21 consecutive day restraint stress model resulted in impaired memory retention as detected by the MWM and novel object recognition tests, as well as behavioral depression measured by SI index and FST, which were restored by 4 weeks of regular treadmill running. These results suggest that this experimental paradigm is valid for exploring the mechanisms underlying improvement or prevention of chronic stress-induced behavioral deficits in memory processing and mood-related illnesses using regular exercise. 

Calbindin d28k, a calcium binding protein, contributes to intracellular Ca2+ homeostasis by buffering excess intracellular calcium and maintaining intracellular Ca2+ levels to prevent depletion ^22^^-^^23^. Calbindin d28k protein expression in hippocampal CA1 cells was markedly reduced by chronic stress, and this decrease was attenuated by exercise (Fig. 1C). This suggests that chronic stress is related to disrupted intracellular calcium homeostasis, and that this process is affected by exercise. Several studies have suggested that calcium channel-dependent depolarization and kinases such as CaMKs play a crucial role in synaptic plasticity in chronic stress-induced behavioral deficits, and that the effects of exercise work through Ca2+/Calmodulin-dependent Kinase II-dependent BDNF and CREB induction to alter synaptic plasticity ^24^^-^^26^. Previous studies support these results and have shown that disrupted calcium homeostasis and signaling by chronic stress may be overcome by regular exercise. 

Lower field excitatory postsynaptic potential (fEPSP) slopes and reduced GluA1 expression in hippocampal CA1 neurons have been observed following chronic stress ^13^. Although synaptic Ca2+ entry occurs predominantly through NMDARs, activity-dependent synaptic Ca2+ currents by AMPARs are also critical for synaptic plasticity, for example during hippocampal NMDAR-dependent LTP ^13^^-^^14^. Arc induction takes place as part of the second wave of molecules that are modulated by c-fos for late-LTP and memory consolidation, and contributes to AMPAR trafficking, synaptic strengthening, and long-term neuronal plasticity at excitatory synapses ^4^^,^ ^27^. Our results, along with previous findings, led us to assess Arc expression in hippocampal CA1. Hippocampal CA1 Arc expression was profoundly enhanced by chronic stress, regardless of exercise regimen, while nuclear localization of Arc was significantly increased by chronic stress, and reverted to the basal state following exercise (Fig. 1D). Recent evidence from in vivo and in vitro studies has demonstrated that a rapid increase in cytoplasmic Arc protein slowly translocates into the nucleus where it associates with nuclear promyelocytic leukemia (PML) and contributes to AMPAR trafficking and homeostatic plasticity in stimulated neurons ^10^^-^^11^. To elucidate the direct relationship between AMPA receptor activity and nuclear localization of Arc protein under chronic stressful condition, CX546, an ampakine that produces delayed desensitization of AMPA receptors, AMPAR-mediated increases in amplitude, and duration of fast, excitatory transmission ^28^, was injected into mice subjected to chronic stress. Nuclear Arc levels in hippocampal CA1 neurons were augmented by stress. This effect was attenuated with CX546 treatment, with corresponding normalization of behavior. Nuclear PML bodies contribute to transcription and mRNA export, and nuclear Arc expression reduces GluR1 transcription and abnormal homeostatic response to increased activity ^1^^,^ ^12^. Based on previous studies, the increase in nuclear Arc protein attenuates the surface trafficking of AMPA receptors by reducing GluR1 levels, which produces AMPAR-mediated downregulation of dendritic-wide homeostatic scaling under maladaptive conditions such as chronic stress. On the contrary, regular exercise is believed to prevent chronic stress-related neurophysiological and behavioral changes. In addition, we suggest that the quantification of nuclear translocation of Arc in hippocampal CA1 cells could act as a biomarker for stress-related behavioral deficits. 
